# Therapeutic impact of low amplitude high frequency whole body vibrations on the osteogenesis imperfecta mouse bone^☆^

**DOI:** 10.1016/j.bone.2013.01.023

**Published:** 2013-04

**Authors:** Maximilien Vanleene, Sandra J. Shefelbine

**Affiliations:** Department of Bioengineering, Imperial College London, London, SW7-2AZ, UK

**Keywords:** Whole body vibration, Osteogenesis imperfecta disease, *oim* mouse model, Bone morphology, Bone formation, Bending properties

## Abstract

Osteogenesis imperfecta (OI) is characterized by extremely brittle bone. Currently, bisphosphonate drugs allow a decrease of fracture by inhibiting bone resorption and increasing bone mass but with possible long term side effects. Whole body mechanical vibrations (WBV) treatment may offer a promising route to stimulate bone formation in OI patients as it has exhibited health benefits on both muscle and bone mass in human and animal models. The present study has investigated the effects of WBV (45 Hz, 0.3 g, 15 minutes/days, 5 days/week) in young OI (*oim*) and wild type female mice from 3 to 8 weeks of age. Vibration therapy resulted in a significant increase in the cortical bone area and cortical thickness in the femur and tibia diaphysis of both vibrated *oim* and wild type mice compared to sham controls. Trabecular bone was not affected by vibration in the wild type mice; vibrated *oim* mice, however, exhibited significantly higher trabecular bone volume fraction in the proximal tibia. Femoral stiffness and yield load in three point bending were greater in the vibrated wild type mice than in sham controls, most likely attributed to the increase in femur cortical cross sectional area observed in the μCT morphology analyses. The vibrated *oim* mice showed a trend toward improved mechanical properties, but bending data had large standard deviations and there was no significant difference between vibrated and non-vibrated *oim* mice. No significant difference of the bone apposition was observed in the tibial metaphyseal trabecular bone for both the *oim* and wild type vibrated mice by histomorphometry analyses of calcein labels. At the mid diaphysis, the cortical bone apposition was not significantly influenced by the WBV treatment in both the endosteum and periosteum of the *oim* vibrated mice while a significant change is observed in the endosteum of the vibrated wild type mice. As only a weak impact in bone apposition between the vibrated and sham groups is observed in the histological sections, it is possible that WBV reduced bone resorption, resulting in a relative increase in cortical thickness.

Whole body vibration appears as a potential effective and innocuous means for increasing bone formation and strength, which is particularly attractive for treating the growing skeleton of children suffering from brittle bone disease or low bone density pathologies without the long term disadvantages of current pharmacological therapies.

## Highlights

► Whole body vibration (WBV) therapeutic impacts on bone were investigated in young osteogenesis imperfecta mice (*oim*). ► WBV beneficial impact on the cortical bone morphology of both femur and tibia in the wild type and *oim* mice ► Bone volume fraction was improved in the proximal tibia of *oim* vibrated mice. ► WBV has beneficial impact on bending properties in wild type vibrated mice but only a positive trend in *oim* mice. ► Bone apposition is not significantly improved in the mid-tibia cortical and proximal tibia trabecular bone in the *oim* vibrated mice.

## Introduction

Osteogenesis imperfecta (OI or brittle bone disease) is a hereditary disease which results in extreme bone fragility. Mutation of the genes coding for collagen type 1 (col-1) is the main cause of OI, resulting in a quantitative or qualitative alteration of col-1 production. This leads to extremely active bone remodelling, disorganized woven bone tissue, reduced trabecular and cortical bone mass and degraded bone mechanical properties [1]. There is currently no direct cure for OI and only symptomatic treatments are available, such as physiotherapy to increase postural strength, surgery to correct bone deformation and bisphosphonate treatment. OI patients treated with bisphosphonates, which reduce the bone resorption, have shown an increase in bone mass and a reduction of fracture and pain [2,3]. Such pharmacologic treatments are now commonly used on children (sometime extremely young) during long periods (2–5 years) with the rationale to maximize the impact on a growing skeleton. However, some concerns have been raised about the equivocal efficiency on the fracture reduction [4,5], the accumulation of those long life drugs and the impact of inhibiting bone remodelling over long periods, which results in the build-up of poor quality, highly mineralized bone [1,6].

It is recognized that the bone tissue is highly responsive to dynamic loading and is able to adapt its architecture and mass to the mechanical loading environment [7–9]. Bone remodelling is sensitive to strain magnitude [10,11], frequency [12,13], number of loading cycles [14], strain rate [15] and rest periods between stimulation [16]. In addition to bone response to high peak strains [17,18], there is also evidence of bone adaptation at low strain but high frequency loading [9,19]. Because high strain exercises in patient suffering from OI may result in fracture, high frequency low amplitude whole body mechanical vibration (WBV) is an attractive low-impact and drug-free approach to stimulate bone formation. The therapeutic impact of WBV treatment has been observed on muscle strength, motion, posture and bone density in various osteopenic populations: young women [20,21], post-menopausal women [22–25] or children with disabling conditions like cerebral palsy [26] or with OI [27] but no effect has been observed on healthy adults [28]. However more investigations are required to confirm the impact of WBV on bone mass and to identify the most efficient vibration parameters and the most responsive target population [29–33].

Numerous studies have investigated the influence of WBV on bone formation using a large variety of animal models (sheep, rat, mouse) [34–37], age (growing, young or old adults) [38–40], vibration frequency (from 20 to 90 Hz) [41–43], maximum peak acceleration (from 0.1 to 3 g) [43,44], treatment duration (from 10 to 30 min) and treatment length (from 2 weeks to 1 year). A significant osteogenic effect was observed in the trabecular bone of both the femoral condyle and tibial metaphysis of adult sheep (1 year treatment, 30 Hz, 0.3 g) [35,36]. In adult mice, an osteogenic response to WBV is observed in the tibial metaphysis with a non-dose dependent response to acceleration (5 weeks treatment, 45 Hz, 0.1, 0.3 and 1 g) [44]. An influence of the mouse genotype was observed: the osteogenic response to WBV inversely correlated to the low (C57Bl/6J), medium (BALB/c) or high (C3H) bone density of the mouse strain (2 to 3 weeks treatment, 45 Hz, 0.25 g) [37]. An age effect was also observed with no WBV effect on aged BALB/c mice bone and low effects on adult mice (5 weeks treatment, 90 Hz, 0.3 and 1.0 g) [40], while 8 week-old growing mice exhibited a positive response in trabecular and cortical bone [38,39]. Investigations of WBV as a treatment for osteoporosis have shown a positive impact on ovariectomized rats with greatest increase in bone mass at high frequencies [34,41,43] while other investigation reported only an impact on cortical bone [42] or no substantial impact [45]. These variable results suggest a more complex involvement of the hormonal system in the mechano-sensitivity of bone to WBV. Interestingly, a positive osteogenic response to “limb vibration” in the absence of weight-bearing has been observed, suggesting an additional mechano-transduction pathway than pure bone strain [9,46].

Previous WBV studies on both patients and animals indicate that vibration is most effective in young growing bone and low density bone. Therefore WBV treatment may offer a promising route to non-invasively stimulate bone formation in OI children. The objectives of the present study were to investigate the effects of WBV on the cortical and trabecular bone formation in growing mice suffering a severe form of osteogenesis imperfecta (*oim* mice).

## Materials and methods

### Animals breeding and whole body vibration (WBV) protocol

All animal experiments followed the British Home office and institutional guidance (project license 70/6852). 24 Homozygous wild type (B6C3Fe-a/a-+/+) and 24 homozygous *oim* (B6C3Fe-a/a-oim/oim) female mice were bred. Due to a procollagen α2 gene recessive mutation, homozygous *oim* mice produce abnormal homotrimeric collagen type I (Col1-(α1)_3_) which results in a phenotype mimicking the human type III osteogenesis imperfecta (small body weight, skeletal deformities and brittle bones) [47]. Starting at 3 weeks of age (just after weaning), 12 mice from each genotype group (vibrated groups: Wild vib and *oim* vib) were placed into a custom built WBV transparent plastic cage for 15 min per day, 5 days in a week during 5 weeks. The cage was vibrated vertically at a frequency of 45 Hz and a peak acceleration of ± 0.3 g. This vibration regimen was demonstrated to be osteogenic on young growing mice [38,39]. The vibration cage had 8 slots (10 ∗ 10 cm each so that 8 mice could vibrate simultaneously) and was mounted on a linear electromagnetic actuator (LAL95-015-70F linear actuator and LAC-1 controller, SMAC Europe Ltd., UK). The linear actuator provided a sinusoidal vertical movement and was force-controlled by a custom made LabVIEW program (NI Corporation Ltd., USA) via a laptop computer and a digital acquisition card (NI USB-6211 multifunction DAQ, NI Corporation Ltd., USA). The actuator was powered by a generator (HY3005D-2, Rapid Electronics Ltd., UK). The acceleration was monitored via an accelerometer (DE-ACCM3D, Dimension Engineering LTD, USA) fixed in the middle of the vibrating cage and the force of the actuator was operator-tuned to obtain a maximum peak acceleration of ± 0.3 g. 12 mice from each genotype group were also placed into the vibrating cage but not subjected to the mechanical vibration (sham groups: Wild sham, *oim s*ham). The mice's body weights were recorded during the 5 weeks of vibration treatment.

The mice were injected intraperitoneally with a calcein solution (20 mg/kg) at 10 and 3 days before sacrifice in order to assess bone apposition [48]. Mouse sacrifice was performed by CO_2_ asphyxia and the mouse tibiae and femora were dissected and cleaned of soft tissues. The right bones were stored in gauze soaked with phosphate buffered solution (PBS) and frozen at − 18 °C. The left bones were fixed in 4% formalin-phosphate buffered solution overnight, rinsed with PBS and stored in 70% ethanol at 4 °C.

### 3D bone morphology analyses

**Fig. 1:**
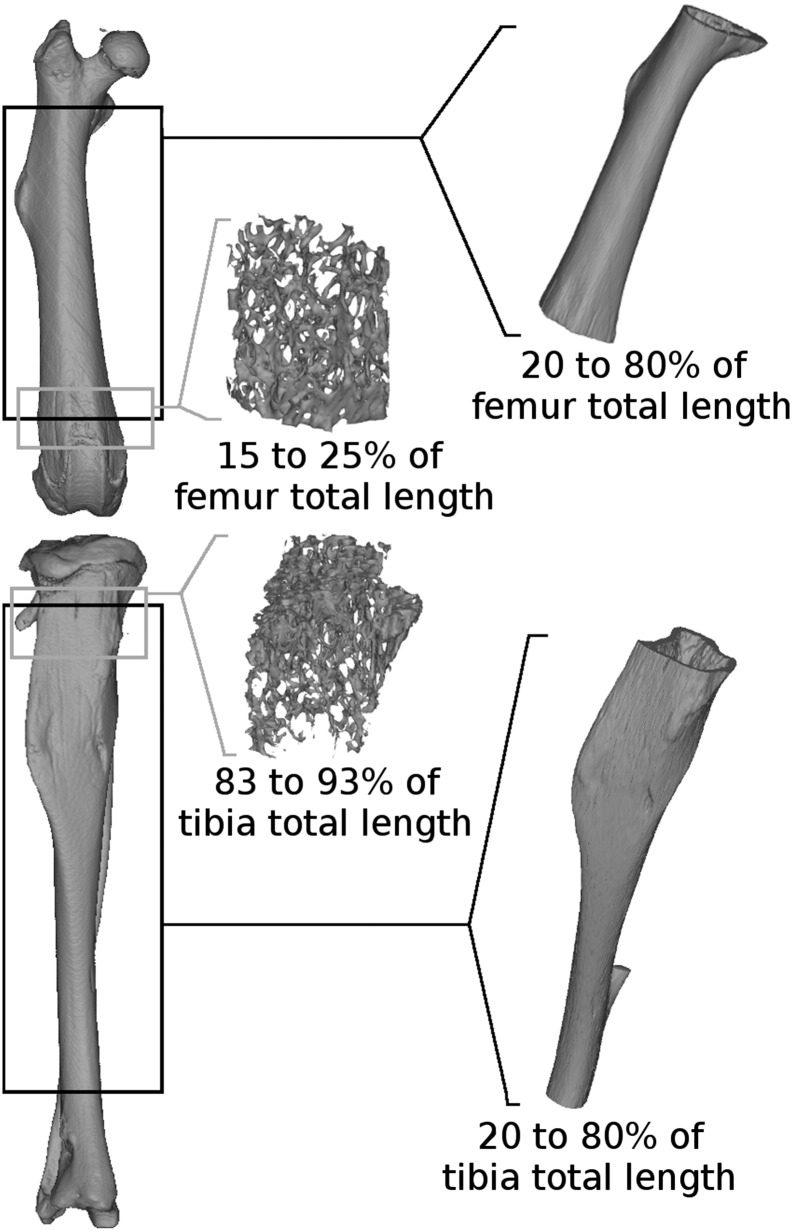
Illustration of the regions of cortical and trabecular bone investigated for the morphology analyses on the micro computed tomography images obtained from the mice femur and tibia.

Right tibiae and femora were scanned using a micro-computer tomography scanner (Metris X-Tek HMX ST 225 CT System) with a 10 μm voxel resolution (80 to 120 kV, 140 μA, 500 μs integration time). Trabecular and cortical bone morphology was analysed in the femur and the tibia using the open source ImageJ software and BoneJ plugin [49]. The cortical bone morphology was analysed (every 10 slices) between 20% and 80% of the femur total length (%TL distal to proximal) and 20% to 90%TL of the tibia after segmenting out the trabecular bone (see Fig. 1). Cortical parameters analysed were as follows: cross section area (CSA, mm^2^), minimum and maximum moment of inertia (I_min_, I_max_, mm^4^) and mean cortex thickness (CtTh, mm). Trabecular bone was analysed (every slice) between 15 and 25%TL in the femur distal metaphysis and between 83 and 93%TL in the tibia proximal metaphysis (see Fig. 1). The trabecular bone was separated from the cortical bone by manually drawing a contour in the proximal tibia while, in the distal femur, an elliptical region of interest (length/width ratio of 1.5) was drawn and replicated every slice. Trabecular bone parameters analysed were as follows: trabecular bone surface (BS, mm^2^), trabecular bone volume on total volume (BVTV), mean trabeculae thickness (TbTh, mm) and mean trabeculae space (TbSp, mm).

### Three point bending mechanical testing

After CT scanning, right femurs were tested until fracture by three-point bending using a standard materials testing machine (5866 Instron, Instron, Norwood, MA, USA). Femurs were placed on their posterior side on two supports separated by 9 mm and were loaded in the anterior-posterior direction at the mid-diaphysis with a deflection rate of 50 μm/s. Force–deflection curves were analysed with a custom program (Matlab, MathWorks Inc, MA, USA) to measure the bending stiffness (S: slope of the linear elastic deformation), the yield force (F_yield_, limit between the elastic and plastic deformation) and ultimate force (F_ult_, maximum force sustained) and the total work to fracture (mJ). The bone elastic modulus E (MPa), ultimate stress σ_ult_ (MPa) and yield stress σ_yield_ (MPa) were calculated using the standard beam theory [50] and the mid femur cross-section dimensions (anteror posterior diameter and medial lateral moment of inertia) measured from the μCT scanner data.

### Histomorphometry of un-decalcified tibia bone

**Fig. 2:**
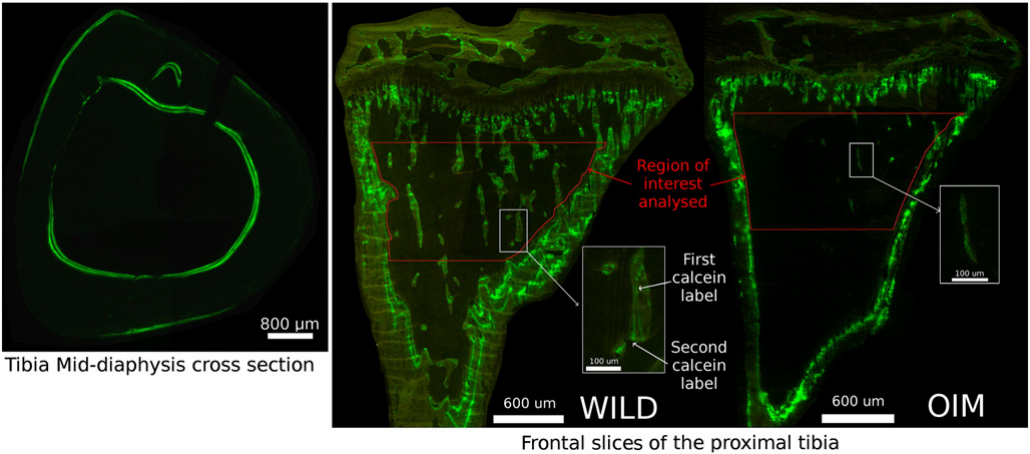
Calcein labels observed by FITC fluorescence light microscopy (10 ×) in the tibia mid-diaphysis cross-section of a wild type vibrated mouse (left) and in the frontal slices of the proximal tibia of a wild type (middle) and *oim* (right) vibrated mice. The red box represent the trabecular bone region of interest (between 200 and 1200 μm under the growth plate) analysed from the proximal tibia.

Left tibiae (5 tibiae per group) were first dehydrated at 4 °C in successive 1 h acetone bath of increasing purity (70, 90 and 2 times in 100% acetone) then infiltrated 3 to 7 days into a destabilized methyl-methacrylate (dMMA) solution with 10% dibutylphthalate (DBP) and 0.05% of benzoyl peroxide (BPO). After infiltration, tibiae were then laid down on prepared polymerized MMA base in individual glass vials and cured in a dMMA solution with 15% DBP and 2% BPO at 37 °C for three days. After removing the cured specimens from the vials, tibiae were cut transversally at the mid diaphysis with a low speed saw (IsoMet® 1000 Precision Saw, Buehler, UK). Distal tibia halves were used to cut a 200 μm mid-diaphysis cortical bone cross-sections which were ground and polished until a thickness of roughly 50 μm was reached. Meanwhile, the proximal tibia halves were sliced in the frontal plan with a Leica 2255 microtome (5 μm thickness) and three slices (separated by 100 μm) were chosen at the middle of the tibia. Mid-diaphyseal cross sections and proximal tibia slices were imaged (10 ×) using a fluorescent microscope (Zeiss Axioplan microscope and Leica DFC 310FX camera) with a fluorescein iso-thio-cyanate filter (480 nm excitation (cyan), 530 nm emission (green)). Bone apposition was analysed using ImageJ software following classical histomorphometry techniques [51]: mineralizing surface on bone surface (MS/BS), mineral apposition rate (MAR, μm/days) and bone formation rate (BFR, μm/day). The tibia metaphyseal trabecular bone was analysed in a 1000 μm long region of interest starting 200 μm under the mineralized front of the growth plate (see Fig. 2). In the mid-diaphysis tibia cross sections, bone apposition was analysed in both the endosteum and the periosteum (see Fig. 2).

### Statistics

Cortical bone morphology μCT scan data were analysed using multi-factor multi-parameter analysis of variance (MANOVA) with vibration treatments (vibrated, sham), mice genotype (wild, *oim*), and position within the diaphysis (20, 30, 40, 50, 60, 70, 80% TL) as factors. Data were then analysed with wild type and oim groups separated, followed by an analysis of each position within the diaphysis individually.

The final mouse body weight, the femur and tibia total length, the trabecular bone μCT morphology data and the three-point bending mechanical data were analysed using a 2-way ANOVA with mice genotype (wild, *oim*) and vibration treatments (vibrated, sham) as factors. Genotype groups were then tested separately.

Histomorphometry data were analysed using non-parametric Mann and Whitney tests. All statistical tests were performed using SPSS 19.0 software with a significance level of 5%.

## Results

### Mouse body weight and hindlimb bone total length

When the genotype groups were tested together, the vibration treatment did not significantly affect the final body weight or the femur and the tibia total length (TL) (p = 0.084, p = 0.12 and p = 0.078 respectively). However, when genotype groups were split, the body weight and the tibia total length were significantly greater in the vibrated wild type group compared to those of the sham wild type group (p = 0.001 and p = 0.046 respectively) but the femur length exhibited no difference (p > 0.05). In the *oim* group, no significant differences were found for the three parameters (p > 0.05 for all).

### Micro CT analysis of cortical bone morphology

Vibration treatment had a significant effect on the cortical morphology parameters (CSA, CtTh, I_max_, I_min_) in the femur and tibia of both wild type and *oim* animals when all the position within the tibia diaphysis were considered (percentage of total length (%TL)). In the wild type group, vibration treatment increased the cross section area (p = 0.026) and the mean cortical thickness (p < 0.001) in the tibia and increased CSA (p = 0.016); I_min_ (p = 0.014) and CtTh (p = 0.001) in the femur. In the *oim* mice group, all cortical parameters showed significant increases between vibrated and sham mice for the femur (CSA: p < 0.001, I_min_: p = 0.008, I_max_: p = 0.012, CtTh: p < 0.001) and for the tibia (CSA: p < 0.001, I_min_: p = 0.012; I_max_: p = 0.019, CtTh: p = 0.001).

**Fig. 3:**
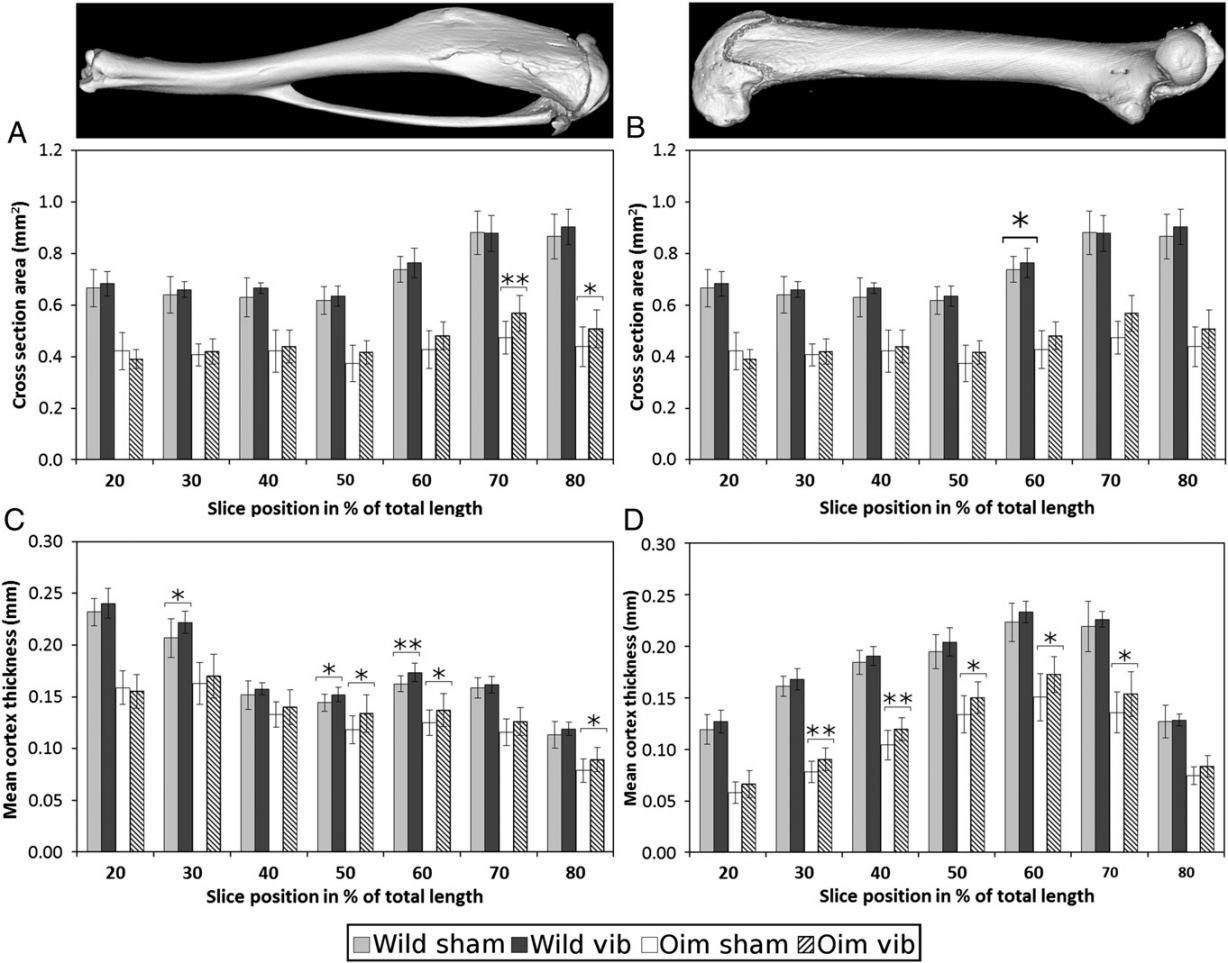
Cortical bone cross section area and mean thickness (mean and standard deviation) measured along the diaphysis of the tibia (A, C) and femur (B, D) of the wild type and *oim* mice (* p < 0.05, ** p < 0.01). Cortical mean thickness was significantly greater in the *Oim* vibrated mice in the proximal tibia and the femur mid-diaphysis. Cross section area was also found significantly greater in the proximal tibia of the oim vibrated mice. Wild vibrated mice exhibited also greater cortical thickness in the tibia mid-diaphysis. In the femur only a tendency is observed but not significant. A tendency toward greater value of cross-section area was also observed in the tibia and femur of the wild type vibrated mice but only significant at 60% of the femur total length.

In the Fig. 3, the differences observed for CSA and CtTh between the vibrated and sham mice are displayed for each of the positions along the tibia (Figs. 3a and b) and femur (Figs. 3c and d). In the femur of the *oim* vibrated mice, mean CtTh exhibited a significant increase for the central portion of the diaphysis (30-70%TL) while the wild mice exhibited a significant increase of CSA at 60%TL (p = 0.045). In the tibia, *oim* vibrated mice exhibited a significant increase of CtTh and CSA at the proximal end of the diaphysis (50-80%TL) while wild type vibrated mice show a significant increase of the mean cortical thickness at various positions (30, 50 and 60% TL).

### Micro CT analysis of trabecular bone morphology

**Fig. 4:**
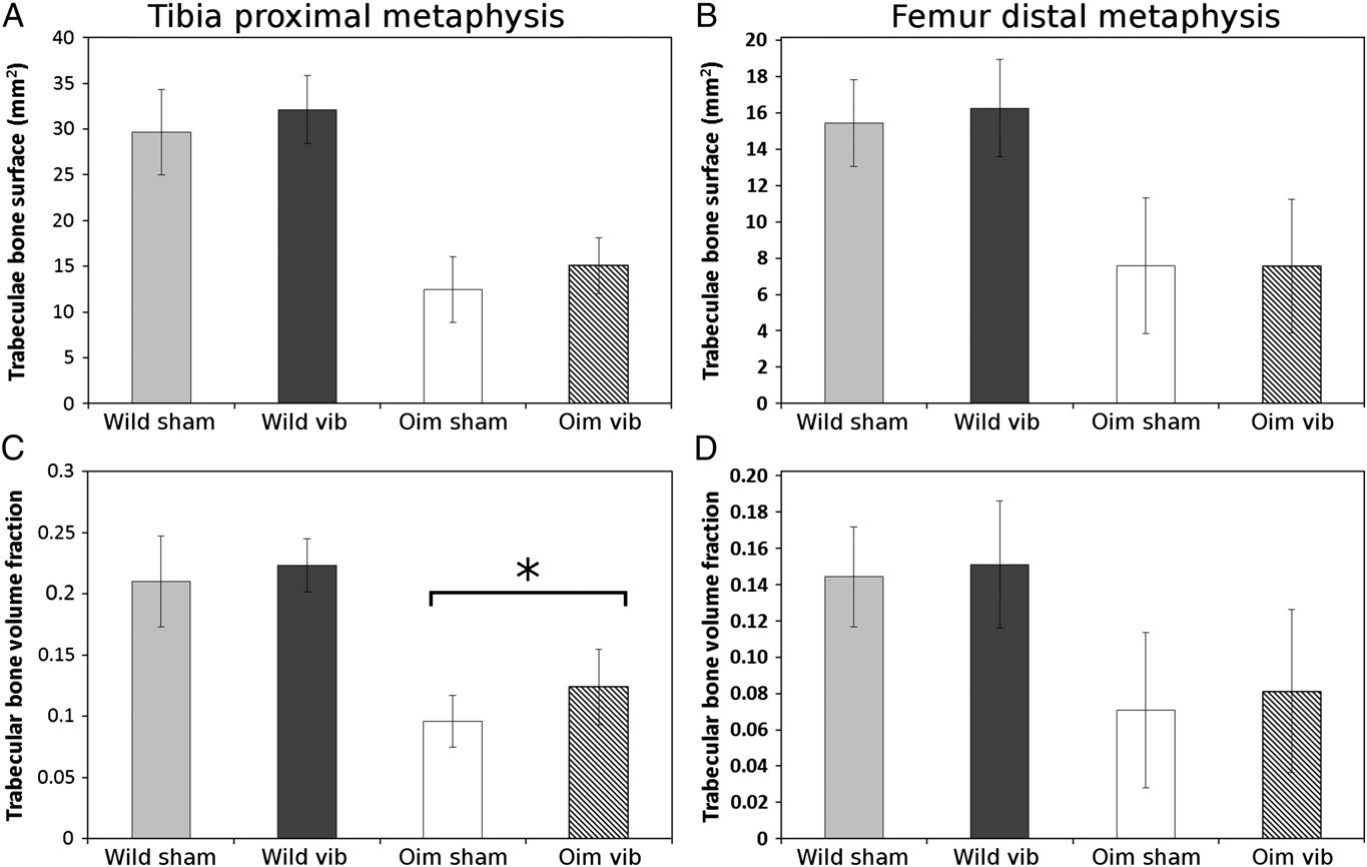
Trabecular bone surface (mm^2^) and bone volume fraction measured in the tibia proximal metaphysis (A, C) and femur distal metaphysis (B, D) of the wild type and *oim* mice. (* p < 0.05, ** p < 0.01). In the tibia, bone volume fraction was significantly greater in the vibrated group for the *oim* mice but not for the wild type mice. In the femur, no significant difference was found between vibrated and sham groups for both parameters. *Oim* vibrated mice exhibited a significantly higher bone volume fraction while wild type mice showed only a non-significant trend. In the femur, no significant difference were found.

In the proximal tibial trabecular bone, a significant difference was observed between vibrated and sham groups. Bone surface and bone volume fraction were significantly increased in the vibrated group (p = 0.03 and p = 0.017 respectively) but not the trabecular thickness and spacing (p > 0.05). When genotype group were analysed separately, the wild type group exhibited no significant difference between vibrated and sham mice for all trabecular parameters (p > 0.05) (Figs. 4a and b). However, the *oim* vibrated mice exhibited a significant increase of the tibia bone volume fraction (p = 0.019) (Fig. 4b).

In the femur distal metaphysis, no significant differences between vibrated and sham mice were found for the trabecular bone morphology parameters in either wild type or *oim* groups (BS, BVTV, TbTh or TbSp, p > 0.05 in all condition, Figs. 4c and d).

### Three point bending mechanical data

In the wild type group, the vibration treatment had a significant impact on the femur bending stiffness and yield load (p = 0.034 and p = 0.035 respectively) but the other parameters (ultimate load, total work to fracture, ultimate stress, Young's modulus and yield stress) were not significantly different. In the *oim* group, a tendency toward greater values was observed in the vibrated mice for all parameters but did not reach statistical significance as there was a large standard deviation, typical of the *oim* phenotype. The point bending data are summarized in Table 1.

### Tibial cortical bone histomorphometry

In all the mice analysed (both wild type and *oim*, vibrated and sham), bone calcein double labels were clearly defined in both periosteum and endosteum of the tibia mid-diaphyseal cross-sections. Bone apposition parameters (MS/BS, MAR, BFR) were not significantly different in the endosteum and periosteum between the vibrated and sham mice when both genotype groups were considered together (p > 0.05 for all parameter). When the genotypes were considered separately, only the MS/BS of the endosteum in the wild type group was significantly increased (p = 0.036) in the wild type group while all other parameters were not significantly different. In the *oim* group, only a non-significant trend toward higher MAR and BFR values was observed in both endosteum and periosteum. Cortical bone histomorphometry data are summarized in Table 2.

### Tibial trabecular bone histomorphometry

In the wild type mice group, morphology of the trabecular bone was well developed with numerous trabeculae and clearly visible calcein double labels. In the *oim* mice, the trabeculae were scarcely present with unclear calcein labels and very few or no visible double labels. No significant differences were found between vibrated and sham mice in the wild type group. In the *oim* group, no statistically significant difference was observed between the vibrated and sham mice. Tibia trabecular bone histomorphometry data are summarized in the Table 2.

## Discussion

In the present study, whole body vibration (WBV) treatment improved the trabecular and the cortical bone morphology during the growth in very young *oim* mouse hind limbs. In the femur, this improvement of the cortical bone morphology correlates with a trend toward an increase of the mechanical properties observed during the three point bending. However the heterogeneity of the *oim* phenotype resulted in large standard deviations as previously reported [52] and the increase in mechanical integrity was not sufficient to reach statistical significance.

In the vibrated wild type mice, the osteogenic effect of WBV on the cortical bone morphology was apparent when the full lengths of the femur and tibia diaphysis were considered. This “global” improvement was sufficient to obtain a significant positive impact on the femur rigidity and yield limit during the three point bending test. The improvement of both cortical and trabecular bone compartment in the *oim* mice tibial metaphysis when subjected to WBV is in accordance with the findings of Xie et al. in slightly older but still growing BALB mice [39] and suggests that growing bone may be particularly sensitive to WBV. In addition, we also observed a positive response in the cortical bone of both femur and tibia, indicating that the WBV could be beneficial for both hind limb long bones in *oim* mice. Interestingly, Xie et al. found no change in cortical thickness, but a positive effect on the cross section area, bone marrow area and I_max_ (enlargement of the metaphysis cross section) [39]. Our investigation showed that WBV had a significant influence on the mean cortical thickness and a more “global” effect on other morphological parameters (i.e. significant if all position within the diaphysis are considered), which may be explained by the difference in the growth period observed. In the present study, we vibrated from 3 to 8 weeks, which corresponds with a rapid growth in length; while in Xie et al. [39], mice were vibrated from 8 to 14 weeks, in which slower growth occurs. In the wild type group, a small osteogenic response was also observed, not at a particular location but in the diaphysis as a whole (as shown by the MANOVA) and only in the cortical bone. The difference of effect between *oim* and wild type groups could be explained by the lower “bone mass” (thinner cortex and lower trabecular bone volume fraction) in the oim group. This may increase the response of the bone tissue to the high frequency low amplitude vibrations as it has been observed in low bone mass mice strain by Judex et al. [37]. Because wild type mice have higher bone mass, they may require a different vibration stimulus to trigger a greater osteogenic response [37] and allow a stronger statistical response. The use of a higher frequency might improve the impact of the WBV [41], but increasing the vibration magnitude (acceleration) has been shown to have little to no effect in the mouse model [44]. A recent computational study has proposed a mechanism of the osteogenic impact of the WBV on the trabecular bone based on the stimulation of the bone cells by the fluid shear stress of the bone marrow on the trabeculae surface generated by high frequency loadings [53]. The simulation demonstrated that a lower trabecular bone volume fraction resulted in higher stresses on the trabeculae surface and therefore in increased stimulation of the bone cells. This is in accordance with our results as *oim* mice had a greater response. Considering the differences observed in the intrinsic mechanical properties and mineralization of the bone between wild type and *oim* mice [54], some differences in vibration propagation due to bone material differences in the two groups might also be considered in addition to the impact of bone morphology.

The sensitivity to the WBV treatment was different between the cortical and trabecular compartments. Indeed, most of the investigations of WBV in adult mouse models reported a positive WBV osteogenic impact in only the tibial trabecular bone [44] with no impact on cortical bone [40,46]. Lynch et al. [40] reported no impact of WBV at all in old mice, which may be interpreted as a change in mechano-sensitivity with age. Interestingly, in ovariectomized rat studies, WBV had a beneficial effect on cortical bone [42,43]. Rubinacci et al. [42] suggested that the difference in response of the trabecular and cortical bone compartments may be sex-hormone dependent and inhibited by oestrogen in the cortical compartment. In the present study, the mice were not sexually mature (limited influence of oestrogen) and were actively growing, which could explain the beneficial effects on cortical bone.

The histomorphometry analyses of bone apposition in the *oim* mice exhibited no significant effect in the trabecular or cortical bone. The lack of positive impact on the trabecular bone apposition observed in the *oim* mice (with histology) contrasts with the significant improvement of the trabecular bone volume fraction (found with microCT). This may be explained by a reduction of the osteoclast activity, rather than an increase in osteoblast activity [38,39]. In addition, in the trabecular bone of the *oim* mice, a very high trabecular bone turnover [55,56] resulted most likely in the resorption of the calcein labels leading to an inaccurate measure of bone apposition. Indeed, the calcein double labels were rarely observable in the trabecular bone of *oim* mice but clearly defined in the cortical mid-diaphysis cross-sections. This will impact the reliability of the measurement of the mineral apposition rate (MAR) and therefore the calculation of the bone formation rate (BFR). Future studies will decrease the time between calcein labels to more accurately capture bone formation dynamics and will also investigate the osteoclasts activity.

In the tibia cortical bone of the wild type mice, the significant increase of MS/BS (and trend toward higher bone formation rate) in the endosteum seems to correlate with the significant increase of the cortical thickness observed at 50% of the tibia total length in the μCT analyses. In the oim mice, the improvement observed at 50% of the tibia total length could not be related to change of the bone formation despite a tendency toward greater values in both endosteum and periosteum of the oim vibrated mice (not significant due to large variability). Also, we only measured the bone apposition in one position along the diaphysis and our micro CT analyses have shown some more effects on the proximal tibia. Others have previously shown the impact of WBV on the cortical bone apposition in the proximal tibia [38]. Future work will use a novel 3D histomorphometry technique to investigate a larger volume of the cortical proximal bone.

The present study has demonstrated the osteogenic impact of a whole body vibration treatment in an osteogenesis imperfecta mouse model with cortical thickness and cross-section area increase in both femur and tibia and a trabecular bone volume increase in the tibia. This might lead to improvement of the mechanical bending properties but only a trend was observed in the *oim* group. The low amplitude high frequency WBV treatment has potential as a non-invasive and non-pharmacologic therapy to stimulate bone formation during growth in OI. The fact that an osteogenic impact was observed in the cortical bone compartment is even more attracting as it is the most likely to have a beneficial effect on the bone mechanical function. Such use of the WBV has been clinically observed in the bone of low bone density child population [21,26] and a positive impact of WBV on the muscle was already reported in young OI patients [27]. Further investigations are required to confirm and optimize the osteogenic effects of the WBV (vibration frequency, acceleration or treatment duration and length) in young children and to determine if the beneficial effects would last during adulthood.

## Figures and Tables

**Table 1:** Femur three point bending data obtained from Wild type and *Oim* mice submitted to vibration or sham: ultimate force (F_ult_, N), bending stiffness (S, N/mm), yield force (F_yield_, N), total work to fracture (mJ), ultimate stress (σ_ult_, MPa), Young's modulus (E, MPa), yield stress (σ_yield_, MPa). (Mean and standard deviation; p value from MANOVA test, level of significance = 5%).

	Wild vib		Wild sham			*Oim* vib		*Oim* sham		
	Mean	SD	Mean	SD	p	Mean	SD	Mean	SD	p
F_ult_	13.5	1.8	12.6	0.9	0.154	6.1	1.3	5.4	1.6	0.425
S	77.7	5.8	69.9	10.4	**0.034**	27.3	10.8	21.7	9.7	0.294
F_yield_	8.2	1.1	7.4	0.7	**0.035**	3.6	1.1	3.0	1.2	0.199
Total work to fracture	7.3	2.0	7.7	2.4	0.68	1.7	0.7	1.6	0.6	0.617
σ_ult_	117.2	8.2	120.4	13.7	0.49	100.2	19.9	93.3	21.4	0.694
E	6544	734	6521	1017	0.95	4511	2505	4023	1404	0.707
σ_Yield_	71.8	7.1	70.7	9.2	0.76	59.8	16.5	49.7	14.0	0.188

**Table 2:** Bone apposition results obtained by histomorphometry analyses of the double calcein labels in the tibia mid-diaphysis cross-section and the tibia metaphysis trabecular bone: ratio of mineral surface on bone surface (MS/BS), mineral apposition rate (MAR, μm/day) and bone formation rate (BFR, μm/day). (Mean and standard deviation; p value from Mann and Whitney tests, level of significance = 5%).

Cortical bone		Wild vib		Wild sham			*Oim* vib		*Oim* sham		
		Mean	SD	Mean	SD	p	Mean	SD	Mean	SD	p
Endosteum	MS/BS	0.68	0.07	0.58	0.07	**0.036**	0.69	0.15	0.72	0.18	0.347
	MAR	1.76	0.02	2.02	0.38	0.525	1.26	0.22	1.09	0.24	0.347
	BFR	1.20	0.13	1.17	0.31	0.675	0.86	0.21	0.77	0.22	0.347
Periosteum	MS/BS	0.45	0.08	0.51	0.10	0.754	0.62	0.15	0.57	0.11	0.6
	MAR	1.41	0.41	1.56	0.16	0.117	1.25	0.31	1.09	0.31	0.6
	BFR	0.65	0.24	0.80	0.18	0.173	0.79	0.35	0.59	0.10	0.465


Trabecular bone		Wild vib		Wild sham			*Oim* vib		*Oim* sham		

		Mean	SD	Mean	SD	p	Mean	SD	Mean	SD	p

	MS/BS	0.24	0.03	0.20	0.03	0.095	0.16	0.04	0.18	0.02	0.548
	MAR	2.23	0.29	2.08	0.19	0.548	1.21	0.18	1.50	0.40	0.25
	BFR	0.53	0.09	0.42	0.09	0.095	0.27	0.11	0.29	0.05	0.786
